# The recombinant pea defensin Drr230a is active against impacting soybean and cotton pathogenic fungi from the genera *Fusarium*, *Colletotrichum* and *Phakopsora*

**DOI:** 10.1007/s13205-015-0320-7

**Published:** 2016-02-13

**Authors:** Ariane Ferreira Lacerda, Rafael Perseghini Del Sarto, Marilia Santos Silva, Erico Augusto Rosas de Vasconcelos, Roberta Ramos Coelho, Vanessa Olinto dos Santos, Claudia Vieira Godoy, Claudine Dinali Santos Seixas, Maria Cristina Mattar da Silva, Maria Fatima Grossi-de-Sa

**Affiliations:** 1 Embrapa Recursos Genéticos e Biotecnologia, PqEB, Avenida W5 Norte (Final), PO 02372, Brasília, DF 70770-917 Brazil; 2 Universidade Federal do Rio Grande do Norte, Avenida Senador Salgado Filho 3000, Lagoa Nova, Natal, RN 59078-970 Brazil; 3 Centro Universitário do Distrito Federal, SEP/SUL EQ 704/ A12 904 Conjunto A, Brasília, DF 70390-045 Brazil; 4 Embrapa Soja, Rodovia Carlos João Strass, Distrito de Warta, A10 PO Box 231, Distrito de Warta, PR 86001-970 Brazil; 5 Universidade Católica de Brasília, SGAN 916 Módulo B Avenida W5, Brasília, DF 70790-160 Brazil

**Keywords:** Defensin, *Pisum sativum*, *Pichia pastoris*, *Fusarium tucumaniae*, *Colletotrichum gossypii* var. *cephalosporioides*, *Phakopsora pachyrhizi*

## Abstract

Plant defensins are antifungal peptides produced by the innate immune system plants developed to circumvent fungal infection. The defensin Drr230a, originally isolated from pea, has been previously shown to be active against various entomopathogenic and phytopathogenic fungi. In the present study, the activity of a yeast-expressed recombinant Drr230a protein (rDrr230a) was tested against impacting soybean and cotton fungi. First, the gene was subcloned into the yeast expression vector pPICZαA and expressed in *Pichia pastoris*. Resulting rDrr230a exhibited in vitro activity against fungal growth and spore germination of *Fusarium tucumaniae*, which causes soybean sudden death syndrome, and against *Colletotrichum gossypii* var. *cephalosporioides*, which causes cotton ramulosis. The rDrr230a IC_50_ corresponding to inhibition of fungal growth of *F. tucumaniae* and *C. gossypii* var. *cephalosporioides* was 7.67 and 0.84 µM, respectively, demonstrating moderate activity against *F. tucumaniae* and high potency against *C. gossypii* var. *cephalosporioides*. Additionally, rDrr230a at 25 ng/µl (3.83 µM) resulted in 100 % inhibition of spore germination of both fungi, demonstrating that rDrr230a affects fungal development since spore germination. Moreover, rDrr230a at 3 µg/µl (460.12 µM) inhibited 100 % of in vitro spore germination of the obligatory biotrophic fungus *Phakopsora pachyrhizi*, which causes Asian soybean rust. Interestingly, rDrr230a substantially decreased the severity of Asian rust, as demonstrated by *in planta* assay. To our knowledge, this is the first report of a plant defensin active against an obligatory biotrophic phytopathogenic fungus. Results revealed the potential of rDrr230a as a candidate to be used in plant genetic engineering to control relevant cotton and soybean fungal diseases.

## Introduction

Plant diseases caused by fungi affect a broad range of crops worldwide, being responsible for significant losses and for the decrease in quality and safety of agricultural products. In crop plants, fungi cause more economic damage than any other group of microorganisms, with annual losses estimated at more than US$ 200 billion (Horbach et al. 2011). There are several fungal diseases impacting in tropical areas, which include cotton ramulosis, Asian soybean rust and soybean sudden death syndrome caused by *Colletotrichum gossypii* (South) var. *cephalosporioides* A. S. Costa, *Phakopsora pachyrhizi* H. & P. Syd., and *Fusarium solani* (Mart.) Sacc. f. sp. *glycines* (= *Fusarium tucumaniae* T.) (Aoki et al. 2003), respectively. Currently, there are no cotton or soybean varieties with good agronomic traits, which at the same time present high resistance against these important diseases. In addition, none of these diseases are satisfactorily controlled by fungicides.

To circumvent fungal infection, plants have developed innate immune systems that recognize the presence of pathogens and initiate effective defense responses (Muthamilarasan and Prasad 2013). Therefore, plants produce diverse molecules such as carbohydrates, lipids (Rojas et al. 2014), antimicrobial enzymes (Habib and Fazili 2007), secondary metabolites, (Ahuja et al. 2012; Bednarek 2012) and several defense-related proteins such as chitinases, glucanases, lectins, thionins, and defensins (Carvalho and Gomes 2009).

Defensins, which are pathogenesis-related proteins produced by plants, invertebrate and vertebrate animals, are basic, small (around 50 amino acids, about 5 kDa), cysteine-rich-peptides whose three-dimensional structure presents one α-helix and three antiparallel β-sheets stabilized by an αβ motif (Lay and Anderson 2005; Carvalho and Gomes 2009). Defensins have a broad range of biological activity, including inhibition of α-amylases (Bloch and Richardson 1991; Pelegrini et al. 2008) and proteases (Wijaya et al. 2000; Melo et al. 2002), antimicrobial activity (Ye and Ng 2001; Wong and Ng 2005; de Zélicourt et al. 2007), inhibition of protein synthesis (Mendez et al. 1990, 1996), blockage of ionic channels (Spelbrink et al. 2004) and interference in cell cycle by DNA binding (Lobo et al. 2007).

Transgenic plants expressing defensins have been shown to be an alternative strategy to protect plants against pathogens (De Bondt et al. 1999; Gao et al. 2000; Parashina et al. 2000; Chen et al. 2002, 2006; Park et al. 2002; Chan et al. 2005; Zhu et al. 2007; Anuradha et al. 2008; Choy et al. 2009; Kovalskaya and Hammond 2009; Abdallah et al. 2010; Jha and Chattoo 2010) specially in case there is no source of natural resistance for breeding programs.

The pea (*Pisum sativum*) defensin Drr230a, transgenically expressed in tobacco, has been previously shown to present antimicrobial activity against the entomopathogenic fungus *Trichoderma reesei* and against the phytopathogenic fungi *F. solani*, *F. oxysporum*, *Ascochyta pisi*, *A. pinodes*, *A. lentis*, *Alternaria alternata,* and *Leptosphaeria maculans* (Lai et al. 2002).

Considering the potential of Drr230a to control other relevant fungal diseases in agriculture, in the present study, we describe the expression of the *drr230a* gene in *Pichia pastoris*, the purification of the resulting recombinant Drr230a (rDrr230a) and evaluations of its activity against the impacting cotton and soybean fungi *C. gossypii* var. *cephalosporioides*, *P. pachyrhizi* and *F. tucumaniae*.

## Materials and methods

### Phytopathogenic fungi

The fungal pathogen *F. solani* (Mart.) Sacc. f. sp. *glycines* (= *F. tucumaniae*), which causes soybean sudden death syndrome, was provided by Dr. Leila Costamilan (Embrapa Trigo, Passo Fundo-RS, Brazil). *C. gossypii* (South) var. *cephalosporioides* A. S. Costa, a fungal pathogen that causes cotton ramulosis, was provided by Dr. Nelson Dias Suassuna (Embrapa Algodão, Campina Grande-PB, Brazil). Both fungi were maintained by in vitro cultivation and spore lyophilization.

The *P. pachyrhizi* H. & P. Syd. uredospores, which cause Asian soybean rust, were collected at soybean fields in Londrina-PR, Brazil and maintained in soybean plants under greenhouse controlled conditions at Embrapa Soja, Londrina-PR, Brazil.

### Subcloning of *drr230a* gene into yeast expression vector

The *drr230a* defensin gene (Genebank accession AF525685), provided by Dr. Richard Fobert (National Research Council Canada, Plant Biotechnology Institute), was originally isolated from pea (*P. sativum*) and previously demonstrated to present antifungal activity (Lai et al. 2002). The *drr230a* gene, initially subcloned into a plant transformation vector, provided by Dr Richard Fobert, was amplified by using the primers DRR230a01For (5′ GCC GAA TTC AAC ACA TGT GAG AAC 3′) and DRR230a02Rev (5′ TGG GCG GCC GCT CAA TGA TGA TGA TGA TGA TGG CAG TTT TTA GTA CAC CAA CAG CGA AAG TCA TC 3′) designed to insert *Eco*RI and *Not*I restriction sites at the 5′ and 3′ ends, respectively, to subclone into the *P. pastoris* expression vector pPICZαA^®^ (Invitrogen Co.). Moreover, by using the reverse primer DRR230a02Rev a coding sequence of six histidines (6xHis) was added to the 3′ end of the *drr230a* gene, to enable purification of the corresponding recombinant rDrr230a protein by immobilized metal affinity chromatography (IMAC) and immunodetection with commercial anti-His antibodies. The amplicon comprising the *drr230a* gene with 6xHis-tag was subcloned into the *Eco*RI and *Not*I sites of pPICZαA^®^ in frame with the α-factor secretion peptide signal, downstream of the alcohol oxidase I promoter.

The resulting construct pPICZαA-Drr230a integrity was confirmed by automated ABI sequencing (Perkim-Elmer). The pPICZαA-Drr230a construct was used to transform *P. pastoris* competent cells strain X-33 by using 20 µg of *Sac*I linearized DNA via electroporation (in accordance with the manufacturer). Clones were selected on YPD broth (1 % yeast extract; 2 % dextrose; 2 % peptone, 2 % agar) plates containing zeocin (100 µg/ml) and by PCR analyses. Clones were then cultivated in plates with higher zeocin concentration (500 µg/ml) to increase chances of selecting those with two gene copies in the yeast genome (Vassileva and Chugh 2001).

### Expression of rDrr230a in *P. pastoris* and its purification by immobilized metal affinity chromatography (IMAC)

One positive clone containing the pPICZαA-Drr230a construct was inoculated in 2 ml of YPD broth (1 % yeast extract; 2 % dextrose; 2 % peptone) and incubated over night at 28 °C and 200 rpm (i.e. pre-inoculum). Afterwards, the 2 ml pre-inoculum was diluted in 200 ml of BMG broth (1.34 % de YNB, 1 % de glycerol, 4 × 10^−5^ % Biotin, 100 mM potassium phosphate buffer, pH 6.0) and incubated for 24 h to reach OD_600_ = 20 (i.e. inoculum). Then, those 200 ml inoculum were centrifuged (1500×*g*; 25 °C; 5 min) and the resulting pellet was resuspended in the same volume of BMM broth (1.34 % de YNB, 1 % methanol, 4 × 10^−5^ % Biotin, 100 mM potassium phosphate buffer, pH 6.0) to induce expression of the recombinant protein rDrr230a. During the induced expression, the culture was incubated at 28 °C and 220 rpm agitation for 4 days. Aliquots of the culture supernatant were analyzed by SDS-TRICINE-PAGE 14 % and Western Blot probed with commercial anti-His antibodies (Invitrogen Co.) to detect the His-tag rDrr230a protein (Towbin et al. 1979; Schagger and Von Jagow 1987).

Supernatant from the expression culture was filtered through 0.2 μm membranes (Millex-GV, Millipore), diluted in binding buffer (100 mM sodium phosphate buffer, pH 7.4; 500 mM NaCl) and applied into a His-Trap FF crude column (1.6 × 2.5 cm; 5 ml) (GE Helthcare) previously equilibrated with binding buffer pH 7.4 at 5 ml/min. Binding recombinant rDrr230a protein was eluted with elution buffer (binding buffer containing imidazole to final concentration of 500 mM) and checked for purity by SDS-TRICINE-PAGE 14 % and Western Blot probed with commercial anti-His antibodies (Invitrogen Co.). The fraction containing the recombinant rDrr230a protein was dialyzed, lyophilized and dissolved in deionized water. The N-terminal amino acid sequence of rDrr230a was confirmed by sequencing using Edman’s degradation technique.

### Antifungal bioassay against non-biotrophic phytopathogenic fungi

Bioassays against the soybean pathogenic fungus *F. tucumaniae* and against the cotton pathogenic fungus *C. gossypii* var. *cephalosporioides* were performed in two quantitative assays. The first assay, performed to analyze inhibition of fungal growth, consisted of incubating 10 µl of a solution containing 10^4^ spores/ml with 80 µl of liquid PDB (Potato Dextrose Broth) and rDrr230a to different final concentrations, varying from 0.2 up to 60 ng/µl in the case of inhibition of *F. tucumaniae* growth, and varying from 5 up to 50 ng/µl in the case of inhibition of *C. gossypii* var. *cephalosporioides* growth (assay total volume corresponded to 100 µl per sample). The assay was done in a 96-well microplate and the samples were incubated at 28 °C for 48 h in the dark. The inhibition of fungal growth was measured by optical density in a microplate reader (BIORAD) at 600 nm. All treatments were done in triplicates. Distilled water and hydrogen peroxide (70 µM) were used as negative and positive controls, respectively.

The second assay, performed to analyze inhibition of spore germination, was carried out using 5 µl of a solution containing 10^4^ spores/ml and 15 µl of a solution containing rDrr230a. The samples were incubated at 28 °C for 12 h. The germinated spores were counted in a Neubauer chamber upon observation under an optical microscope (Olympus Optical Co.) within several fields of 100 spores/field. Moreover, representative sample fields were photographed with the same equipment. All treatments were done in triplicates. Distilled water and hydrogen peroxide (70 µM) were used as negative and positive controls, respectively.

### Antifungal bioassay against obligatory biotrophic phytopathogenic fungus

Bioassays against the obligatory biotrophic soybean pathogenic fungus *P. pachyrhizi* were performed in two assays. The first assay was performed to analyze the disease severity caused *in planta* by *P. pachyrhizi* in presence or absence of rDrr230a. This assay was carried out by inoculating half soybean detached leaflets with 150 µl of a solution containing 8 × 10^4^ uredospores/ml (in sterile distilled water containing 0.01 % v/v Tween 20), by brushing. Fourteen minutes afterwards, the same half leaflets were treated, by brushing, with rDrr230a to final concentration 3 µg/µl. The leaflets were then incubated at 24 °C for 14 days in a humid chamber. The severity was evaluated daily. All treatments were done in triplicates. Distilled water was used as the negative control. In addition, 24 h post inoculation, one half leaflet of each treatment was punched with a microtube cap to collect samples for microscopy analyses of uresdospore germination under a Scanning Electron Microscope. For this reason, each collected leaf disk was immediately fixed in glutaraldehyde/paraformaldehyde solution. Afterwards, the leaf disks were further fixed in osmium tetroxide and then in uranyl acetate. Dehydration of the leaf disks was performed in acetone solutions of serially increasing concentrations. Acetone was removed from the leaf disk samples by critical point drying. Finally, the leaf disks were coated with gold by low-vacuum sputter coating, then observed and photographed under Scanning Electron Microscope (Jeol).

The second assay, performed to analyze inhibition of uredospore germination, was carried out by using 50 µl of a solution containing 10^4^ uredospores/ml (in sterile distilled water containing 0.01 % v/v Tween 20) and rDrr230a at final concentration of 3 µg/µl, in a final volume of 100 µl. The assay was done in a 96-well microplate and the samples were incubated at 24 °C in a humid chamber overnight. To end up the assay, lactophenol was added. The germinated uredospores were counted in a Neubauer chamber upon observation with an optical microscope (Olympus Optical Co) within several fields of 100 uredospores/field. Moreover, representative sample fields were photographed with the same equipment. All assays were done in triplicates. Distilled water was used as negative control of inhibition of uredospore germination.

## Results and discussion

### Expression of rDrr230a in *P. pastoris* and its purification by immobilized metal affinity chromatography (IMAC)

**Fig. 1:**
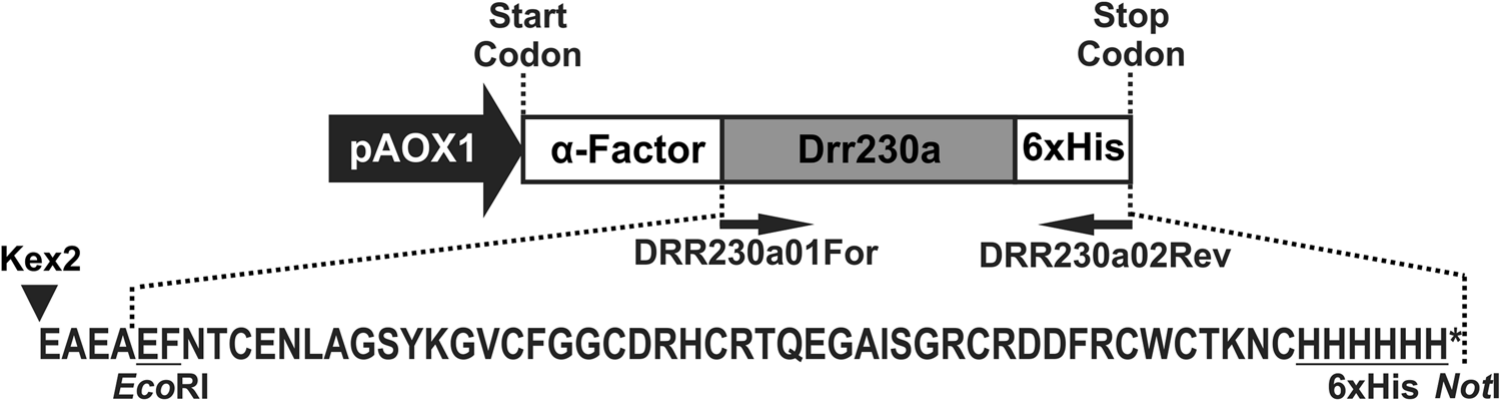
Schematic description of the expression cassette construct designed to express Histidine-tagged recombinant Drr230a in yeast (*P. pastoris*). The *drr230a* gene was subcloned into the yeast expression vector pPICZαA (Invitrogen Co.) under the control of the alcohol oxidase 1 promoter (pAOX1) inducible by methanol. The orientations of the primers DRR230a01For and DRR230a02Rev, including the respective restriction *Eco*RI (resulting in addition of *EF* residues at the recombinant protein N-terminus) and *Not*I cloning sites (not expressed), are indicated by *arrows*. The recombinant protein (sequence depicted above) is expressed in frame with the *Saccharomyces cerevisiae* secretion mating α-factor signal peptide (α-Factor), which is cleaved before protein export at the Kex2 signal cleavage site (Kex2), resulting in the addition of four residues (EAEA) at the N-terminal end of the recombinant protein. A Histidine tag (6xHis; *HHHHHH*), followed by a stop codon (*asterisk*) was included at the C-terminal end of the sequence to facilitate recombinant protein purification by Immobilized metal affinity chromatography

To evaluate the biological activity of rDrr230a against phytopathogenic fungi, the corresponding gene was introduced into the *P. pastoris* expression vector pPICZαA and the rDrr230a protein was expressed under control of the pAOX1 methanol-inducible promoter (Fig. 1). The resulting recombinant protein rDrr230a was secreted due to the α-mating factor signal peptide present in the vector (Fig. 1). A poly-histidine tag (6xHis) was added to the 3′ end of the gene sequence to enable purification of the resulting recombinant His-tagged protein (Fig. 1) through IMAC. *P. pastoris* strain X-33 was transformed with the expression construct and resulting colonies were assayed in plates with high concentration of the selection agent zeocin. Zeocin resistant clones were used in a small-scale expression assay. The clone that presented the highest expression level was chosen to proceed to large-scale production of rDrr230a.

**Fig. 2:**
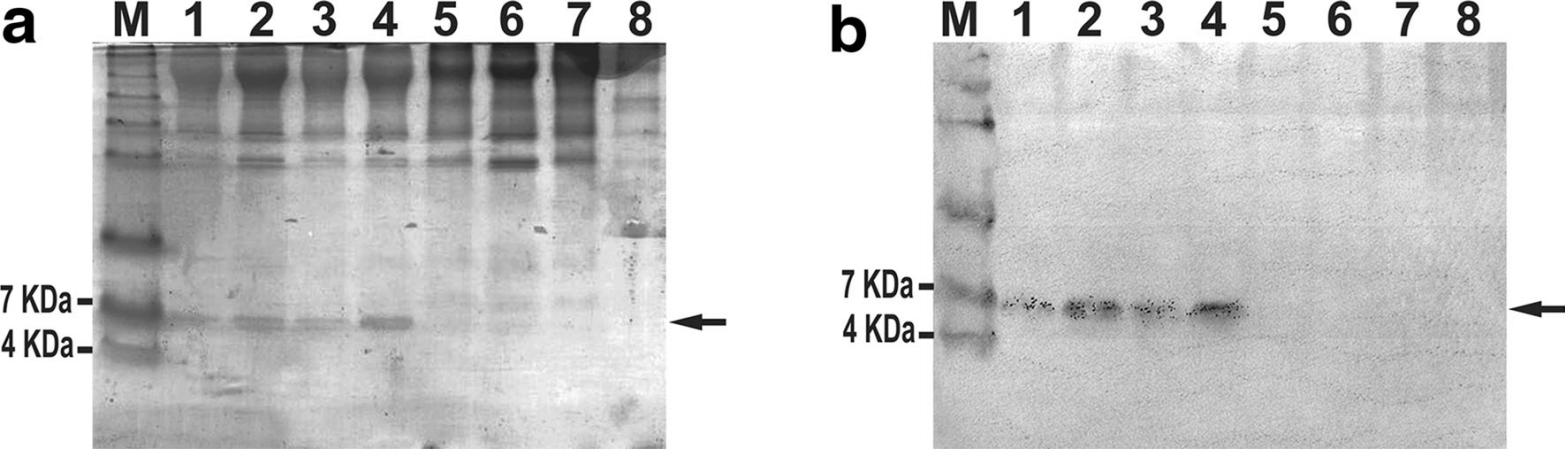
Expression of rDrr230a in *P. pastoris* X-33 cells. **a** SDS-TRICINE-PAGE analysis of the expression of the His-tagged rDrr230a protein from 24 to 96 h after methanol-induction. **b** Western blot analysis of expressed His-tagged rDrr230a protein probed with anti-His antibody (Invitrogen). Molecular mass marker See Blue^®^Plus 2 Prestained Standard, Invitrogen (lane M). Supernatant from *P. pastoris* X-33 culture expressing rDrr230a 24 h (*lane 1*), 48 h (*lane 2*), 72 h (*lane 3*) and 96 h (*lane 4*) post-induction. Supernatant from *P. pastoris* X-33 untransformed culture 24 h (*lane 5*), 48 h (*lane 6*), 72 h (*lane 7*) and 96 h (*lane 8*) post-induction. *Arrows* indicate bands corresponding to the expressed rDrr230a (6.52 kDa)

The recombinant protein rDrr230a was expressed upon promoter induction with methanol, and untransformed cells were used as negative control sample. Aliquots from the expression set up were collected every 24 h in a time course from 24 h up to 96 h, and analyzed in TRICINE-SDS-PAGE. The production of rDrr230a occurred at 24 h, remaining until 96 h, and rDrr230a presented the expected molecular size of 6.52 kDa (Fig. 2a, b).

**Fig. 3:**
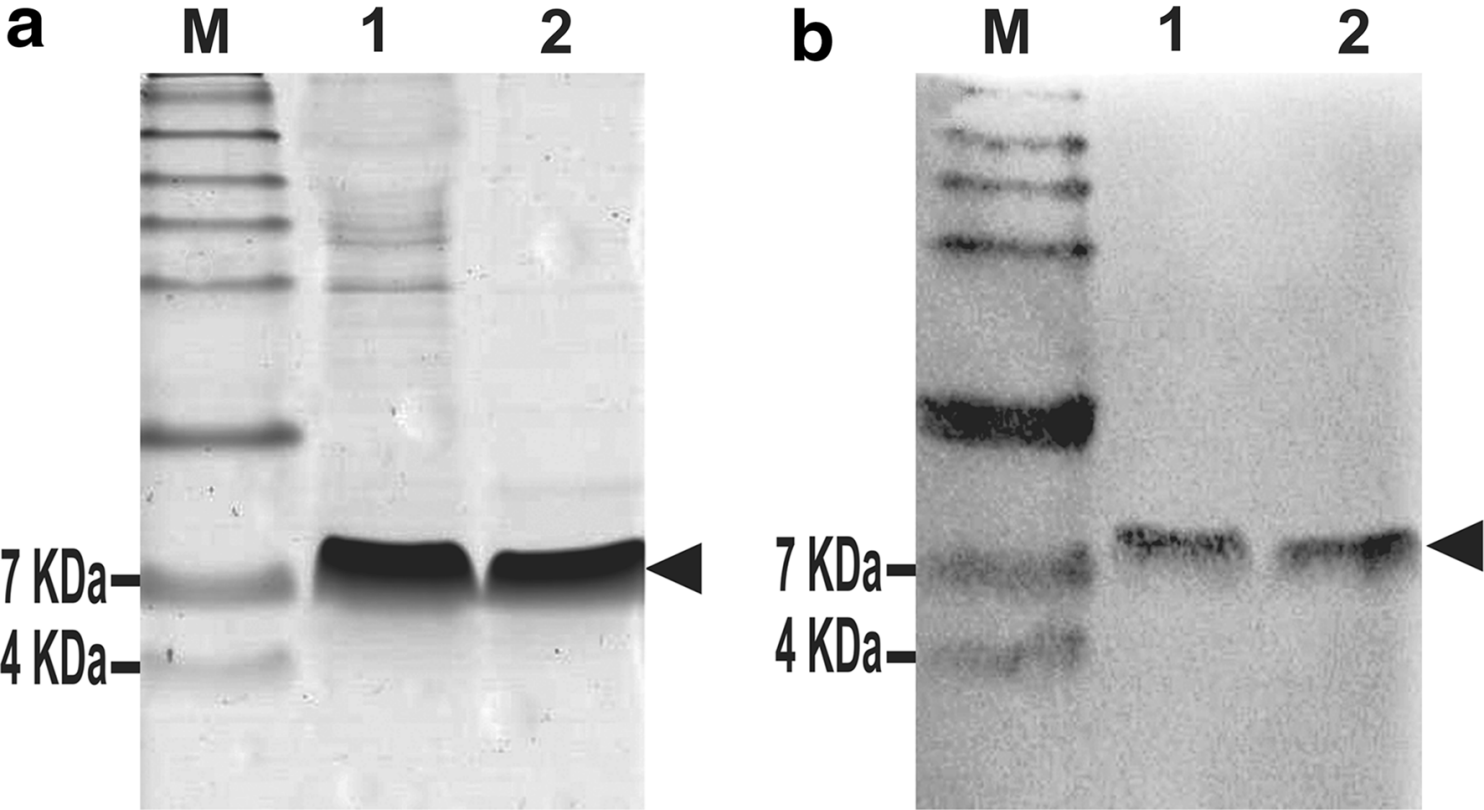
Purification of rDrr230a expressed in *P. pastoris* X-33 cells. rDrr230a was purified by immobilized-metal affinity chromatography (IMAC). **a** SDS-TRICINE-PAGE analysis of the purification of rDrr230a. **b** Western blot analysis of purification of rDrr230a, probed with anti-His antibody (Invitrogen). Molecular mass marker See Blue^®^Plus 2 Prestained Standard, Invitrogen (*lane M*). Supernatant from *P. pastoris* X-33 culture expressing rDrr230a 96 h after methanol-induction (*lane 1*). Eluate fraction from IMAC, corresponding to *P. pastoris*-expressed rDrr230a 96 h after methanol-induction (*lane 2*). *Arrows* indicate bands corresponding to the purified rDrr230a (6.52 kDa)

The supernatant, containing the secreted rDrr230a, was directly submitted to purification by IMAC. The eluted fractions were analyzed by TRICINE-SDS-PAGE 14 % and Western blot probed with anti-His antibody, confirming the expression of a recombinant protein comprising 6.52 KDa (Fig. 3a, b). The N-terminal amino acid sequence of rDrr230a was confirmed by Edman’s degradation sequencing, which revealed additional amino acids corresponding to leftovers of the Kex2 cleavage site (EAEA) and *Eco*RI site (EF) at the N-terminus (Fig. 1). Considering as well the prediction of amino acid sequence based on nucleotide sequence, the resulting protein sequence of the His-tagged recombinant rDrr230a (57 residues) is EAEA EF NTC ENL AGS YKG VCF GGC DRH CRT QEG AIS GRC RDD FRC WCT KNC HHHHHH, as shown in Fig. 1. Quantification of purified rDrr230a, done by 2D-Quant method (Amersham), indicated a recombinant protein recovery estimated in 3.5 mg/l.

### rDrr230a activity against non-biotrophic and obligatory biotrophic phytopathogenic fungi

**Fig. 4:**
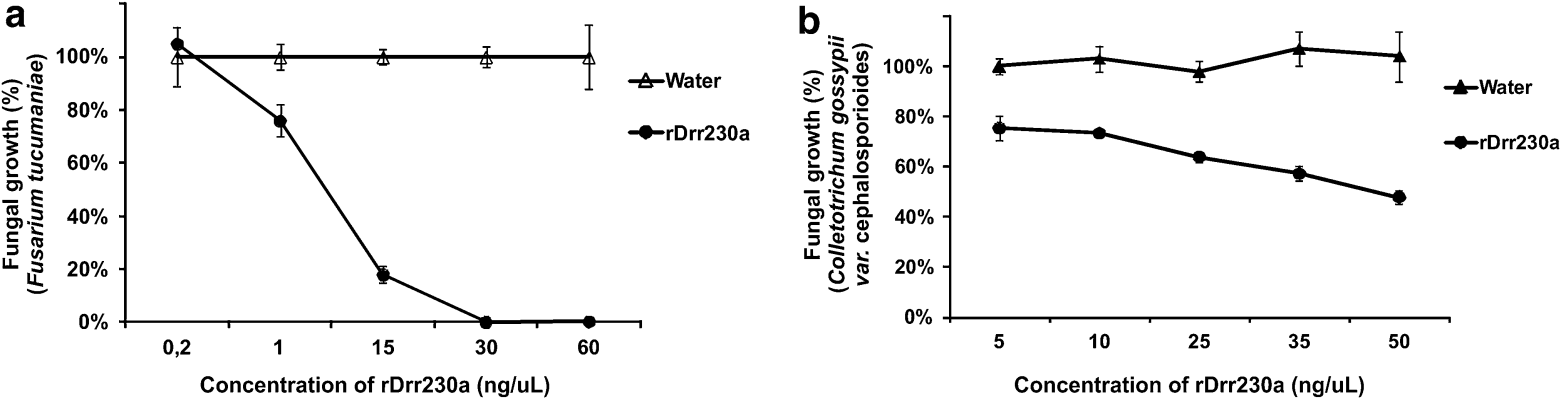
Inhibition of fungal growth of the non-biotrophic phytopathogenic fungi *F. tucumaniae* and *C. gossypii* var. *cephalosporioides* by rDrr230a. Bioassays were performed in microplate wells in liquid medium for 48 h. The data are mean ± standard deviation (*n* = 3). **a** Curve of inhibition of *F. tucumaniae* growth upon incubation with rDrr230a in a concentration range varying from 0.2 ng/µl up to 60 ng/µl (0.03 µM up to 9.20 µM). Calculated IC_50_ against *F. tucumaniae* corresponds to 7.67 µM (50 ng/µl). **b** Curve of inhibition of *C. gossypii* var. *cephalosporioides* growth upon incubation with rDrr230a in a concentration range varying from 5 up to 50 ng/µl (0.77 µM up to 7.67 µM). Calculated IC_50_ against *C. gossypii* var. *cephalosporioides* corresponds to 0.84 µM (5.5 ng/µl)

To analyze inhibition of fungal growth upon incubation with the defensin, spores of the non-biotrophic fungi *F. tucumaniae* and *C. gossypii* var. *cephalosporioides* were incubated with rDrr230a in microplate wells in liquid medium for 48 h. This bioassay was performed by using a concentration range of rDrr230a varying from 0.2 ng/µl up to 60 ng/µl (0.03 µM up to 9.20 µM) to analyze inhibition of *F. tucumaniae* growth and a range varying from 5 up to 50 ng/µl (0.77 µM up to 7.67 µM) in the case of *C. gossypii* var. *cephalosporioides* (Fig. 4). Based on the resulting curves of inhibition of fungal growth of both fungi upon incubation with rDrr230a, the calculated IC_50_ against *F. tucumaniae* was 7.67 µM (50 ng/µl; Fig. 4a) and against *C. gossypii* var. *cephalosporioides* corresponded to 0.84 µM (5.5 ng/µl; Fig. 4b), indicating that rDrr230a is moderately efficient against *F. tucumaniae* and highly potent against *C. gossypii* var. *cephalosporioides*.

**Fig. 5:**
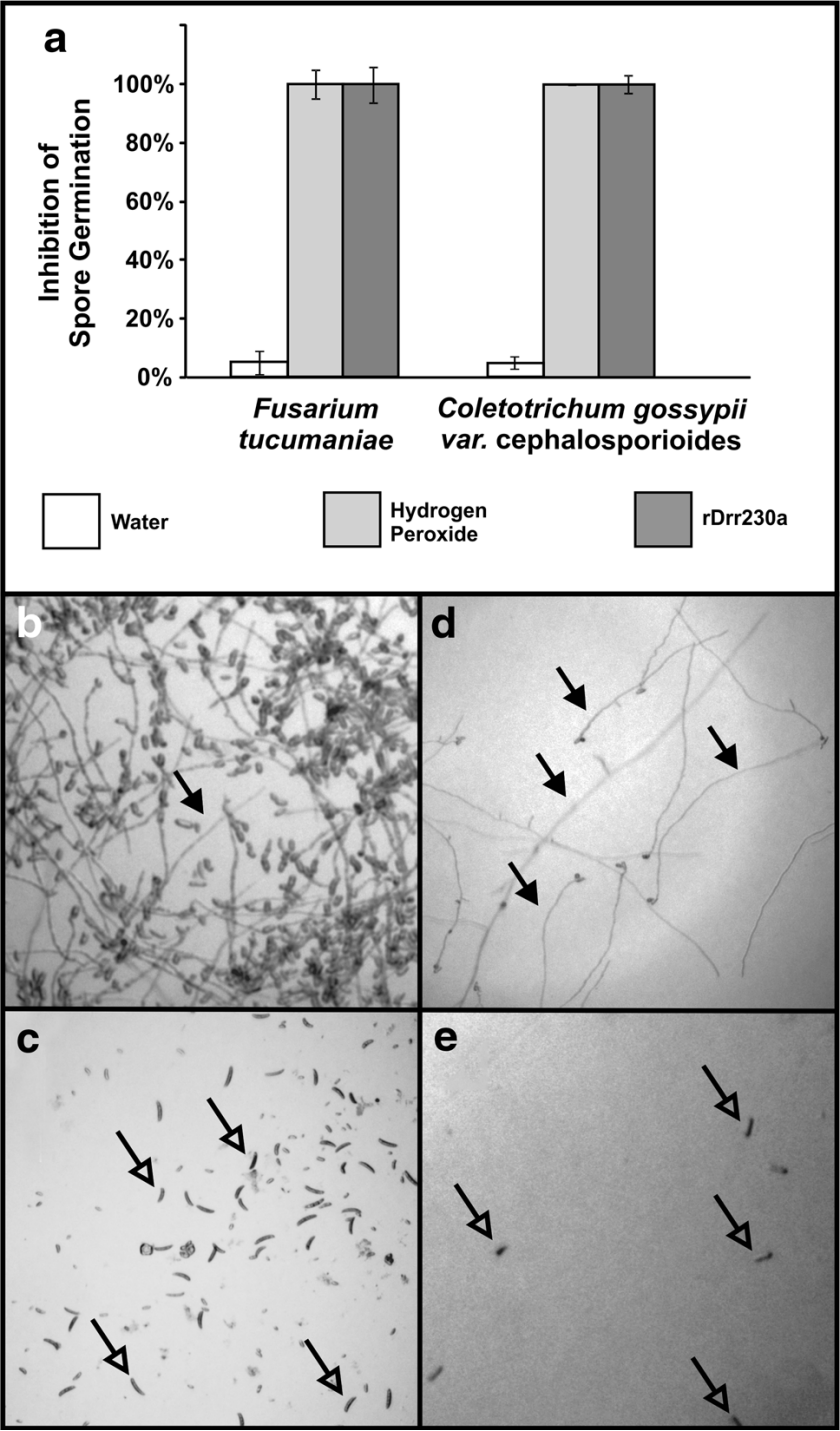
Inhibition of spore germination of the non-biotrophic phytopathogenic fungi *F. tucumaniae* and *C. gossypii* var. *cephalosporioides* by rDrr230a. Bioassays were performed in microplate wells in liquid medium for 12 h. **a** Percentage of the inhibition of spore germination of *F. tucumaniae* and *C. gossypii* var. *cephalosporioides* upon incubation with rDrr230a. Fungal spores were incubated either with rDrr230a at 25 ng/µl, water or hydrogen peroxide at 70 µM. The data in **a** are mean ± standard deviation (*n* = 3). **b** Representative image of *F. tucumaniae* germinating spores (*closed arrows*) under water incubation. **c** Representative image of *F. tucumaniae* non-germinating spores (*open arrows*) under rDrr230a incubation. **d** Representative image of *C. gossypii* var. *cephalosporioides* germinating spores (*closed arrows*) under water incubation. **e** Representative image of *C. gossypii* var. *cephalosporioides* non-germinating spores (*open arrows*) under rDrr230a incubation

To further investigate the deleterious effect of rDrr230a upon both fungi, the activity of rDrr230a against spore germination of *F. tucumaniae* and *C. gossypii* var. *cephalosporioides* was tested at 25 ng/µl (3.83 µM) 12 h post incubation, in a similar microplate setup as previously described. At concentration 25 ng/µl (3.83 µM) the spore germination of both *F. tucumaniae* and *C. gossypii* var. *cephalosporioides* was inhibited in 100 % (Fig. 5a, c, e) despite the fact that the spores of both fungi germinated normally under incubation in water (Fig. 5a, b, d). These observations demonstrate that rDrr230a affects the fungal development of *F. tucumaniae* and *C. gossypii* var. *cephalosporioides* from the very beginning, i.e. since the spore germination.

The *P. pastoris* system was suitable to produce active rDrr230a (Figs. 2, 3) as shown by biological activity of the resulting recombinant protein against *F. tucumaniae* and *C. gossypii* var. *cephalosporioides* (Fig. 4). There are several other reports corroborating that yeast expression systems are adequate to produce active antimicrobial peptides rich in sulfide-bridges, such as defensins. For instance, *P. pastoris* yeast expression systems were successfully used to produce active plant defensins such as the corn defensin PDC1 (Kant et al. 2009), the dimeric defensin SPE10 (Song et al. 2005), the pea defensin Psd1 (Almeida et al. 2001), the sugar beet defensin AX2 (Kristensen et al. 1999), the mungbean defensin VrD1 (Jrchen et al. 2004) and the wild tobacco defensin NmDef02 (Portieles et al. 2010).

Besides expression of plant defensins in yeast systems, there are reports as well of expression of plant defensins in bacteria, in all cases resulting in production of bioactive peptides against various fungi, such as the wild indigo defensin TvD1 (Vijayan et al. 2008), the pepper defensin J1-1 (Seo et al. 2014) and the corn defensin PDC1 (Kant et al. 2009). Nevertheless, when expressed in *E. coli* as compared to the *P. pastoris* system, PDC1 expressed in yeast was more efficient to inhibit growth of *F. graminearum* than the same protein expressed in bacteria (Kant et al. 2009), as it is expected, since yeast is an eukaryotic system, what favors correct cysteine-bond formation, protein correct folding and consequently protein full activity (Demain and Vaishnav 2009; Fickers 2014).

Concerning recombinant plant defensins tested against phytopathogenic fungi from the *Colletotrichum* genus, Seo et al. (2014) demonstrated activity of the defensin J1-1, expressed in bacteria, against *C. gloeosporioides* (IC_50_ 8.3 μM). The defensin SPE10, expressed in yeast, also presented antifungal activity when tested towards the same fungal species (Song et al. 2005), i.e. *C. gloeosporioides*, despite being less potent than J1-1 (IC_50_ >20 μM). Interestingly, rDrr230a is considerably more potent than J1-1 and SPE10 against a target fungus from the *Colletotrichum* genus (*C. gossypii* var. *cephalosporioides*) (IC_50_ 0.84 μM), what represents around tenfold increase in antifungal potency. This implies that the recombinant rDrr230a expressed in yeast was correctly folded and therefore was biologically active.

In what concerns activity against the *Fusarium* genus, rDrr230a (IC_50_ 7.67 μM) was shown to be more effective than the defensin PDC1 produced in bacteria (IC_50_ 30 μM), though being as effective as the same recombinant defensin expressed in yeast (IC_50_ 7.5 μM; Kant et al. 2009). Moreover, rDrr230a (IC_50_ 7.67 μM) was more effective than the defensin SPE10 (IC_50_ > 20 μM), but less effective than the recombinant defensins VrD1 (IC_50_ 1.0 up to 3.4 μM against different *F. oxysporum* isolates), NmDef02 (IC_50_ 1.0 μM against *F. oxysporum*) and TvD1 (IC_50_ < 5.0 μM against both *F. oxysporum* and *F. moniliforme*) against *Fusarium* species (Jrchen et al. 2004; Song et al. 2005; Vijayan et al. 2008; Portieles et al. 2010).

**Fig. 6:**
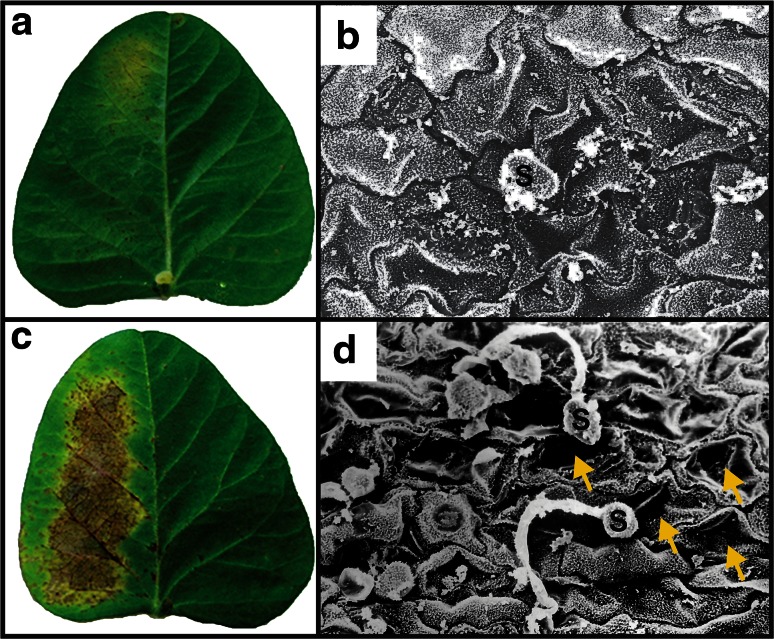
Plant disease severity (Asian soybean rust) caused by the obligatory biotrophic phytopathogenic fungus *P. pachyrhizi* upon incubation with rDrr230a. **a**, **c** Representative half detached soybean leaflets inoculated with *P. pachyrhizi* uredospores either in presence rDrr230a at 3 µg/µl (**a**) or absence of rDrr230a (**c**) 14 days post inoculation. **b**, **d** Sampled areas from inoculated detached leaflets fixed at 1 day post inoculation and analyzed by scanning electron microscopy. Representative micrographs depict no uredospore (S) germination onto well preserved plant epidermal cells when rDrr230a is present (**b**), whereas there is normal uredospore (S) germination onto damaged epidermal plant cells (*yellow arrows*) in absence of rDrr230a (**d**)

**Fig. 7:**
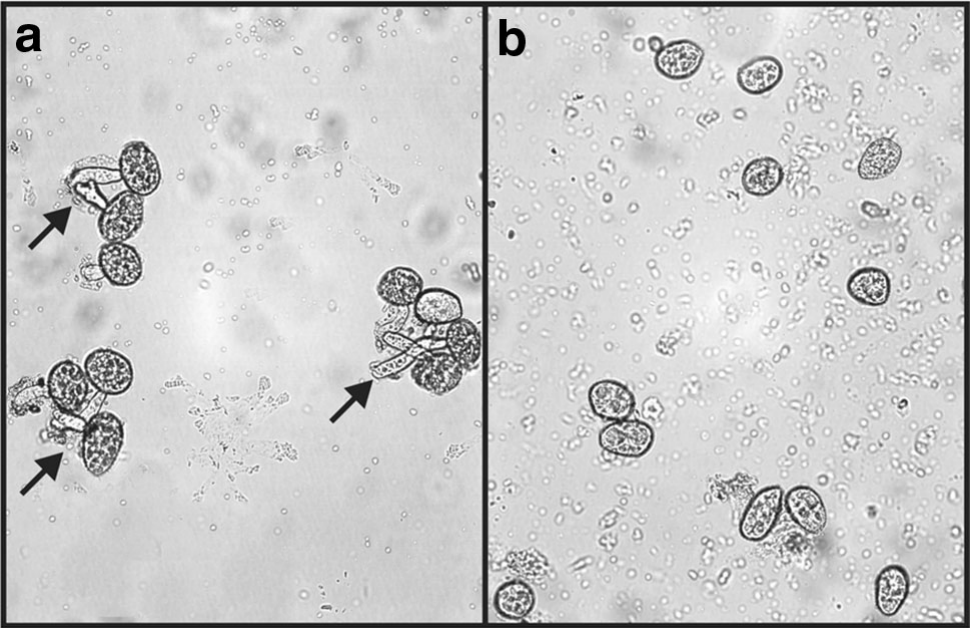
Inhibition of spore germination of the obligatory biotrophic phytopathogenic fungus *P. pachyrhizi* by rDrr230a. Bioassays were performed in microplate wells in liquid medium for 12 h. **a** Representative image of *P. pachyrhizi* germinating spores (*arrows*) under water incubation. **b** Representative image of *P. pachyrhizi* non-germinating spores under incubation with rDrr230a at 3 µg/µl

As *P. pachyrhizi* is an obligatory biotrophic phytopathogenic fungus, it is not feasible to assess rDrr230a in vitro activity against its growth. Therefore, the impact of rDrr230a upon *P. pachyrhizi* infection was assessed by *in planta* bioassays. For that reason, soybean detached leaflets were inoculated with *P. pachyrhizi* uredospores either in presence or absence of rDrr230a and the disease severity was evaluated daily for 2 weeks, by using a scale varying from 0 % up to 100 % severity (Godoy et al. 2006). rDrr230a at 3 µg/µl (460.12 µM) resulted in no disease, i.e. 0 % severity, as compared to average 80 % rust severity in the absence of the recombinant protein, 14 days post inoculation (Fig. 6a, c). A leaf disk sample of each treatment was fixed at 1 day post inoculation for scanning electron microscopy analyses. The corresponding micrographs depict representative images of no uredospore germination onto well preserved plant epidermal cells when rDrr230a is present, whereas there is normal uredospore germination onto damaged epidermal plant cells in absence of rDrr230a (Fig. 5b, d). Moreover, inhibition of *P. pachyrhizi* uredospore germination was evaluated in vitro, under similar setup as previously evaluated for *F. tucumaniae* and *C. gossypii* var. *cephalosporioides*. In this case, rDrr230a inhibited 100 % spore germination of *P. pachyrhizi* at 3 µg/µl (460.12 µM) (Fig. 7). Despite the considerably high concentration used that inhibited in vitro *P. pachyrhizi* spore germination and decreased *in plant* disease severity, to our knowledge, this is the first report of a plant defensin being active against a obligatory biotrophic phytopathogenic fungus.

The antifungal effect of wild type Drr230a protein (also named as DRR206) was firstly reported by Wang and Nowak (1999) who showed that transgenic canola (*Brassica napus*) expressing Drr230a was active against the fungus *L. maculans*, which causes blackleg disease on Brassica crops. Wang and Fristensky 2001 additionally demonstrated that Drr230a expressed in transgenic canola was effective against the phytopathogenic fungi *Rhizoctonia solani* (soil-borne pathogen that causes various plant diseases in a wide host range such as collar rot, root rot, damping off and wire stem) and *Sclerotinia sclerotiorum* (that causes white mold in a wide host range of plants).

In 2002 Lai and cols showed that leaf extracts from transgenic tobacco plants expressing Drr230a were active against the entomopathogenic fungus *T. reesei* and the phytopathogenic fungi *F. solani*, *F. oxysporum* (both *Fusarium* species that cause various diseases in plants and have extremely broad host range), *A. pisi*, *A. pinodes*, *A. lentis* (all *Ascochyta* species that cause blight in legume crops), *A. alternata* (that causes leaf spot in numerous plant hosts) and *L. maculans* (that causes blackleg on *Brassica* spp.). It is not possible to compare the potency of Drr230a against the target fungi herein tested and other affected fungi previously described because of lack of previous literature IC_50_ data available. Nevertheless, the previously reported antifungal activities of Drr230a expressed in transgenic plants is compatible with the herein presented data. Furthermore, the present data demonstrates that the recombinant rDrr230a expressed in yeast retained its antimicrobial property, interestingly against different fungal species as previously tested and with remarkable high potency against *C. gossypii* var. *cephalosporioides*.

The effect of extra residues upon antimicrobial activity of recombinant defensins is a case-wise issue. For instance, Kant et al. (2009) showed a minor deleterious effect of 6xHis-tag upon the antifungal activity of the defensin PDC1 against *F. graminearum*. Nevertheless, the activity of the defensin Psd1 against *Aspergillus niger*, a saprophytic fungus that causes post-harvest black mold in fruits and vegetables, was abolished in the presence of the four leftover residues from the Kex2 cleavage (EAEA) at the N-terminal of the recombinant protein (Cabral et al. 2003). In spite of the fact that rDrr230a expressed in yeast has extra residues, due to the Kex2 cleavage site left over (EAEA) and *Eco*RI site (EF) at the N-terminus and the presence of 6xHis-tag at the C-terminus (Fig. 1), the antifungal activity of rDrr230a was considerably retained, what is indicated by the remarkable high antimicrobial efficiency (IC_50_ 0.84 µM) against *C. gossypii* var. *cephalosporioides* (Fig. 4).

There are several reports of plant defensins transgenically expressed in plants for protection against pathogenic fungi (De Bondt et al. 1999; Gao et al. 2000; Parashina et al. 2000; Chen et al. 2002, 2006; Park et al. 2002; Chan et al. 2005; Zhu et al. 2007; Anuradha et al. 2008; Choy et al. 2009; Kovalskaya and Hammond 2009; Abdallah et al. 2010; Jha and Chattoo 2010), including the Drr230a defensin, which is object of the present study (Wang and Nowak 1999; Wang and Fristensky 2001; Lai et al. 2002). Nonetheless, to our knowledge, this is the first report of a plant defensin active against *P. pachyrhizi*, *F. tucumaniae* and *C. gossypii* var. *cephalosporioides*, which are relevant tropical phytopathogenic fungi of soybean and cotton crops. Since there is neither satisfactory chemical control nor natural sources of resistance against *P. pachyrhizi*, *F. tucumaniae* and *C. gossypii* var. *cephalosporioides*, these results point to rDrr230a as a promising tool for the development of transgenic soybean and cotton plants against this set of impacting tropical diseases.

## Conclusions

The successful expression and purification of the defensin rDrr230a by using *P. pastoris* yeast system, as well as the antimicrobial activity of rDrr230a against impacting cotton and soybean pathogenic fungi was demonstrated in this article. Notably it was demonstrated the inhibition of fungal growth and spore germination of the economically important phytopathogenic fungi *F. tucumaniae*, *P. pachyrhizi* and *C. gossypii* var. *cephalosporioides*, which cause soybean sudden death syndrome, Asian soybean rust and cotton ramulosis, respectively. The data herein presented indicate moderate activity of rDrr230a against *F. tucumaniae* and high potency against *C. gossypii* var. *cephalosporioides*. rDrr230a also drastically decreased the severity of Asian soybean rust. Moreover, rDrr230a affects the development of the three target fungi tested since spore germination. These results reveal the potential of rDrr230a for plant genetic engineering to control relevant tropical fungal diseases of cotton and soybean, for which there are neither satisfactory chemical control nor resistant varieties commercially available.
